# Evaluation of anesthesiology residents in the diagnosis and control of malignant hyperthermia: comparison of three scenarios of realistic simulation ‒ a cross-sectional controlled study

**DOI:** 10.1016/j.bjane.2025.844615

**Published:** 2025-03-28

**Authors:** Mariana F.L. Neville, Victor Guimarães de Almeida, Pamela Vieira Andrade, Joilson Moura Santos, Masashi Munechika, David Ferez, Mary dos Santos Silva, Leonardo Ayres Canga, Ana Cristina Tripoloni, Thierry Girard, Helga Cristina Almeida da Silva

**Affiliations:** a Universidade Federal de São Paulo, Centro de Hipertermia Maligna do Departamento de Anestesiologia, Dor e Terapia Intensiva, São Paulo, SP, Brazil; b Universidade Federal de São Paulo, Centro de Simulação, São Paulo, SP, Brazil; c University Hospital Basel, Anaesthesiology, Basel, Switzerland

**Keywords:** High fidelity simulation training, Malignant hyperthermia, Medical education

## Abstract

**Introduction:**

Simulation-based training is particularly beneficial for rare and life-threatening diseases such as Malignant Hyperthermia (MH). In addition, cognitive aids, including flowcharts and checklists, can be used as guidance in crisis, reducing cognitive demand and simplifying patient care. We assessed the technical and non-technical performance of anesthesiology residents when diagnosing and treating a hypothetical case of MH in three different scenarios.

**Methods:**

This was an observational, cross-sectional, and controlled study. Pairs of anesthesiology residents participated in a validated high-fidelity MH realistic simulation in one of three different scenarios: 1) Control (no access to cognitive aids), 2) Poster, or 3) Mobile application. Both poster and mobile application provided a flowchart and information related to MH diagnosis and treatment. Demographic data, perceived stress levels, and technical and non-technical skills were registered and compared among the groups.

**Results:**

Thirty residents (5-pairs for each scenario) participated in the simulations. The mean score in the technical skill survey was significantly higher in the poster and mobile application groups compared with the control group (83 [4.4], 83 [3.8], and 74 [8.2], respectively, ANOVA, p = 0.047). A significantly higher score for non-technical skills was also found for the poster and mobile application groups compared with the control group (55 [2.5], 57 [0.8], 52 [2.1], respectively, ANOVA, p = 0.03).

**Conclusion:**

In a realistic high-fidelity MH simulation, the participants had satisfactory performance regarding technical and non-technical skills. However, the groups with access to cognitive aids achieved better scores, with no difference between the groups with access to the MH poster and the MH mobile application.

## Introduction

Malignant Hyperthermia (MH) is an inherited autosomal dominant disorder typically triggered by halogenated anesthetics and/or succinylcholine. This pharmacogenetic disorder affects the skeletal muscle by causing excessive calcium release from the sarcoplasmic reticulum, leading to a hypermetabolic response.^1^ This generates heat and leads to tachycardia, hypercarbia, hyperthermia, muscular rigidity, hypoxemia, acidosis, arrhythmia, rhabdomyolysis, kidney failure, and hemodynamic instability. MH is linked to at least three genes: RYR1, STAC3, and CACNA1S. Combined with epigenetic and environmental factors, this genetic heterogeneity results in clinical variability that impacts the differential diagnosis of the crisis.^2^^,^^3^ Incidence rates of MH range from 1:15000 in children to 1:50000–100000 in adults under anesthesia.^4^ MH has been historically regarded as a crisis with high morbidity and mortality rates. However, dantrolene was a turning point in the treatment of MH, as it reduced the mortality rates from 80% in the 1960s to 10% in the present day.^5^

Simulation-based training is a method that was initially developed for aviation training programs but has been increasingly adopted in healthcare programs.^6^ Simulation-based training is increasingly employed before healthcare students conduct procedures on real patients.^7^ Simulations are crucial in rare clinical events and crisis contexts. They enable training in a safe environment, where students can develop their technical and non-technical skills ‒ including behavioral, psychomotor, and emotional skills ‒ in both low and high-complexity procedures. In simulations, students can practice repeatedly until they achieve excellence in a given skill. Interacting with teachers when discussing their performance in simulations through guided reflection or debriefing can also benefit students. Therefore, simulation-based training has yielded better results in healthcare training when compared to traditional teaching methods.^8^ Simulation-based training is particularly beneficial for MH treatment training since it is a rare disease, that many anesthesiologists might not encounter in their lifetime, and leads to a crisis with a high mortality rate when not diagnosed and treated immediately.^9^ To provide treatment for the patient with the best chance of success, the healthcare team's response must be immediate, coordinated, and multidisciplinary. Given the low incidence of MH, details about its diagnosis, treatment, and management must be reviewed and reinforced throughout periodic training sessions. A response plan to an MH crisis must be developed to guide the multidisciplinary team in every healthcare service. This plan must be adapted to local circumstances and practiced and improved during periodic simulations.^10^ As a result, healthcare professionals may be able to address real occurrences in a structured and assertive manner.

In addition, cognitive aids, including tools such as flowcharts and checklists, can be used as guidance in crisis. These aids, which can be displayed as posters or software applications, enhance the safety of the assistance provided by the team during rare situations, facilitating access to relevant information. Critical event guidelines, such as the Society of Pediatric Anesthesia's Critical Events Checklists, have been distributed globally via interactive smartphone apps and were rated as “excellent” on the Systems Usability Scale.^11^ As a result, they may reduce cognitive demand and simplify patient care. Even in clinical situations, such as clinical emergencies, cognitive aids were associated with a reduction in the incidence of missed care steps from 43.3% to 11%. Also, the use of cognitive aids was related to decreases in the incidence of errors, increases in the rate of correctly performed steps, and improvement in clinical teamwork skills scores, non‐technical skills score, subjective conflict resolution scores, and the global assessment of team performance.^12^ In anesthesia, cognitive aids have been increasingly adopted in various crisis scenarios, including MH.^13^ Therefore, this study aimed to assess the technical and non-technical (behavioral) performance of pairs of anesthesiology residents when diagnosing and treating a hypothetical case of MH. The case scenario was conducted in a high-fidelity simulator with and without cognitive aids ‒ a written protocol (poster) and a mobile application.

## Methods

This was an observational, cross-sectional, and controlled study. It was approved by the Research Ethics Committee of the Escola Paulista de Medicina of the Universidade Federal de São Paulo under the CAAE number 96412418.0.0000.5505. All participants signed an informed consent form.

Initially, demographic and perceived stress surveys were filled out. The demographic survey included the following variables: age, sex, institution where the respondents obtained their degree, and institution where they served as anesthesiology residents. The perceived stress survey consisted of a visual analogue scale ranging from 0 to 10, where 0 means no stress and 10 means maximum stress, to measure the respondent's perceived stress before, during, and after the simulations.

### Study groups

A total of 15-pairs of anesthesiology residents participated in the MH simulations. Each pair consisted of two residents from the same institution, one in the first year and the other in the third year of their specialization program.

All participants attended a 2-hour class about MH ‒ its characteristics, diagnostic criteria, and treatment ‒ a week before the training session. The residents were introduced to each other and had the opportunity to be acquainted with the poster and mobile application before the day of the simulation. Each pair of residents could only participate in one simulation during the study.

Each pair was included sequentially in the following groups: 1) Control: no access to cognitive aids in the simulation. 2) Poster: access to a poster with a flowchart and information related to MH diagnosis and treatment. The poster was adapted by the research team based on the poster created by the Malignant Hyperthermia Association of the United States (MHAUS) (mhaus.org). 3) Mobile application: access to a mobile application with a flowchart and information related to MH diagnosis and treatment. The application MHApp – Malignant Hyperthermia (Girard Development GmbH) was developed by Thierry Girard and endorsed by MHAUS and the European Malignant Hyperthermia Group (EMHG).

### Scenario

The scenario contained all the necessary equipment for the simulation to be sufficiently realistic, including an operating room, an operating table, an anesthesia workstation, a multi-parameter monitor (for non-invasive blood pressure measurement, continuous electrical cardiac activity, oximetry, capnography, and thermometry), a defibrillator, medicine flasks, venous access and orotracheal intubation tools, complete surgical apparel, and a SimMan Essential high-fidelity simulator (Laerdal Medical, United States). This simulator consists of an interactive mannequin with thoracic movements, auscultation of normal and abnormal sounds (cardiac, respiratory, and gastrointestinal), and pulse palpation. Immediate responses to each intervention can be programmed previously and/or in real-time. Actors of the simulation were undergraduate or graduate students who were previously trained and always played the same roles as surgeon (n = 1), nurse (n = 1), nursing assistant (n = 1), and intensivist (n = 1). Before the simulation with residents, four rehearsal sessions were carried out with four different senior anesthesiologists over 4-weeks.

The residents were informed about the patient's medical history. They also received the predefined results of routine physical examinations conducted before surgery. The simulator was previously programmed with initial parameters set at normal conditions, which were gradually changed according to the symptoms of an MH crisis. These parameters changed as the residents performed diagnoses, requested laboratory tests, and performed effective treatment procedures. The simulation structure was adapted from the scenarios of MH simulation, progression, and assessment proposed by Corvetto & Taekman^4^ and Arriaga et al.^14^ In summary, the patient was a young woman, without a personal or family medical history of adverse events after anesthesia, who underwent an urgent orthopedic surgery, with a full stomach, under general anesthesia. The anesthesia care team was supposed to carry out a rapid sequence induction, followed by maintenance with halogenated anesthetics. The first signs of MH gradually worsened: hypercarbia, tachycardia, muscular rigidity, blood gas with mixed acidosis, hyperkalemia, white-to-violet soda lime, and serum CK (Creatine Kinase) increase. Depending on the effectiveness of the measures adopted by the residents, the parameters either improved or worsened. The scenario was interrupted after approximately 20-minutes and the patient was transferred to an ICU (intensive care unit) physician.

The pairs of residents did not receive specific guidance regarding their role choices. They were free to decide who would be the leader. Before the simulation, the residents received a 5-minute briefing about the procedures to be carried out (venous access, intubation, monitoring, and medication administration) and the simulation scenario (use of anesthesia machine and defibrillator). After the simulation, a 20-minute debriefing was carried out with the residents to discuss their activities and the progress and characteristics of the case and the simulation, followed by a perceived stress survey. The debriefing was conducted by a researcher with experience in both simulation-based training and MH. All simulations and debriefings were recorded.

As the simulated clinical scenario was the same for all the participants, it has a unique MH clinical grading scale value of 78 (hypercarbia: 15, tachycardia: 3, muscular rigidity: 15, acidemia: 10, rhabdomyolysis: 15 (serum CK increase, hyperkalemia), hyperthermia: 15, and fast reversal with dantrolene: 5).^15^ All the components of this scale were discussed during the debriefing because they highlight important elements for characterizing the observed scenario. However, it was emphasized that this scale is not meant to be used during the crisis itself, but afterward, to characterize the clinical presentation. A low value on this scale should not preclude the treatment once the team has raised the diagnosis of MH, as there are paucisymptomatic and even *fruste* forms of MH.

### Assessment of technical skills

Technical skills were assessed using an adapted version of the survey developed and validated by Gaba^16^ (Supplemental Material Table 1). The survey contained a list of activities related to the identification, diagnosis, and treatment of MH: initial MH protocol, dantrolene administration, ventilation/oxygenation, metabolic management, hyperthermia management, and general management. The maximum score for the survey was 95-points. Each activity consisted of various steps. The activities “initial MH protocol” and “dantrolene administration” had one essential item each (respectively, “terminates triggering agent within 1-minute of notifying surgeon or requesting MH box” and “gives dantrolene ≥ 20 mg within 10 min of MH box arrival”). Even if residents performed all activities correctly, if these two essential steps for the management and treatment of the MH crisis were not performed, their technical skill scores would be zero.

### Assessment of non-technical skills

Non-technical (behavioral) skills were assessed using an adapted version of the Line/LOS checklist developed by NASA (National Aeronautics and Space Administration) and the Aerospace Crew Performance Project, which was previously adapted by Gaba et al.^16^ (Supplemental Material 2). This survey assessed 10 behavioral aspects: orientation, investigation, communication, feedback, leadership, group atmosphere, preparation, task distribution, vigilance, and re-evaluation. It also assessed two general behavioral aspects, one related to the leader and the other to the team. Each of the 10 aspects comprised between 4 and 8 items, and the score for each aspect was the average of its item scores. The items were assessed on a scale from 0 (behavior not identified) to 5 (excellent behavior). Therefore, the maximum total score was 60 points.

Technical and non-technical skills were calculated by obtaining the average score provided by two independent observers who watched a video containing the briefing, the simulation scenario, and the debriefing of each pair.

### Statistical analysis and sample size calculation

The normality of the variables was assessed using a Q-Q plot to verify the normality of the residual values. Categorical data are shown as absolute numbers (n) and percentages (%). Quantitative variables are shown as mean ± Standard Deviation (SD) or median (minimun‒maximum). ANOVA and Kruskal-Wallis tests were performed to compare the variables with normal and abnormal distribution, respectively, across the three groups, while a Chi-Squared test was performed to analyze the categorical variables. The software programs JASP 0.18.3 and IBM SPSS Statistics 25 were used to perform the statistical analysis.

The sample size calculation was estimated to compare the percentage of correct answers about MH before/after the intervention (cognitive aids). Based on previous work in our department, for an average increase of 24% (SD = 11.3%) in the percentage of correct answers after educational activities, with an alpha significance level of 5% and statistical power of 80%, four individuals would be needed in each group.^17^

## Results

Among the 30 participants, the majority was male (21/30, 70%), with a median age of 28 (25–33). Most participants (26/30, 86%) had previous experience with high-fidelity realistic simulations either during their Advanced Cardiac Life Support (ACLS) course or as part of their undergraduate program. Only three participants (3/30, 10%) had previous experience with a confirmed or suspected MH crisis during the anesthesiology specialization (Table 1).

**Table 1:** Baseline characteristics of participants.

	MH Poster	MH App	Control	p
Age, mean (SD)	30 (2.1)	27 (1.7)	29 (4.7)	0.196^a^
Male, n (%)	5 (50%)	7 (70%)	9 (70%)	0.568^b^
Previous experience with RS	10 (100%)	9 (90%)	7 (70%)	0.089^b^
Previous experience with MH	1 (10%)	0	2 (20%)	0.224^b^

MH, Malignant Hyperthermia; RS, Realistic Simulation.

a ANOVA or.

b Chi-square.

When examining the perceived stress levels of participants before, during, and after they participated in the simulation, the highest scores were observed during the simulation, with no statistically significant difference detected between the groups at the three moments (Table 2). There was no correlation between stress levels and performance (technical and non-technical) at each time point (before/during/after simulation) (Supplemental Material Table 3).

**Table 2:** Perceived stress levels of participants before, during, and after their participation in the simulation.

	MH Poster	MH App	Control	p^a^
**Before (mean, SD)**	3.7 (3.3)	2.6 (1.6)	3.1 (1.9)	0.6
**During simulation (mean, SD)**	6.1 (1.6)	6.3 (2.1)	6.8 (1.0)	0.63
**After (mean, SD)**	2.7 (1.9)	3.7 (2.4)	3.7 (2.2)	0.51

a ANOVA

The mean score in the technical skill survey was in all groups above 80% of the maximum of 95 points, with a significantly higher value for the MH poster and application groups than for the control group (Table 3). Among the specific items in the survey, the values in the control group were significantly lower for “ventilation and oxygenation” and “hyperthermia management” (Table 4).

**Table 3:** Average score in technical and non-technical skills.

	MH Poster	MH App	Control	All participants	p^e^
**Technical skills**	83 (4.4)^a^	83 (3.8)^b^	74 (8.2)	80 (6.9)	0.047^e^
**Non-technical skills**	55 (2.5)^c^	57 (0.8)^d^	52 (2.1)	55 (2.9)	0.003^e^

Technical skills: adapted version of the survey developed and validated by Gaba et al.^16^

Non-technical skills: adapted version of the Line/LOS checklist developed by NASA (National Aeronautics and Space Administration) and the Aerospace Crew Performance Project, adapted by Gaba et al.^16^

Post-hoc tukey (vs. control):

a p = 0.074

b p = 0.074

c p = 0.044

d p = 0.003

e ANOVA.

**Table 4:** Scores in individual items of technical and non-technical skills.

	MH Poster	MH App	Control	p^a^
**Technical skills**				
Recognition	10 (10‒10)	10 (10‒10)	10 (8‒10)	0.117
Initiation of MH protocol	15 (9‒15)	15 (10‒15)	12 (0‒14)	0.127
Dantrolene administration	30 (29‒30)	30 (30‒30)	30 (30‒30)	0.368
Ventilation and oxygenation	15 (13‒15)	15 (15‒15)	10 (10‒15)	0.022^a^
Metabolic management	6 (3‒6)	6 (5‒6)	6 (5‒6)	0.906
Hyperthermia management	6 (4‒7)	4 (2‒5)	3 (2‒5)	0.033^a^
Miscellaneous management	4 (4‒6)	5 (4‒6)	4 (4‒5)	0.7289
**Non-technical skills**				
Orientation	5 (5‒5)	4.75 (4.75‒5)	4.5 (4‒5)	0.028^a^
Inquiry/assertation	3.75 (3.25‒5)	5 (4.5‒5)	4 (3.25‒4.25)	0.042^a^
Communication	4.8 (4.4‒5)	5 (5‒5)	4 (3.2‒4.4)	0.003^a^
Feedback	5 (5‒5)	4.5 (3.75‒5)	5 (4.75‒5)	0.016^a^
Leadership	4.62 (4.37‒4.87)	5 (5‒5)	4.37 (4.25‒4.75)	0.006^a^
Group climate	5 (4.5‒5)	5 (4.3‒5)	4.5 (4.5‒5)	0.829
Anticipation/planning	4.75 (4.25‒4.75)	5 (4.5‒5)	4.25 (3.5‒4.75)	0.073
Workload	4.25 (3.75‒4.25)	5 (4.5‒5)	4.25 (4‒4.25)	0.006^a^
Vigilance	5 (4.33‒5)	5 (5‒5)	4.66 (4.66‒5)	0.094
Reevaluation	4.75 (4.5‒5)	4 (3.75‒4.5)	4.75 (4.5‒4.75)	0.014^a^
Primary overall	4 (4‒5)	5 (5‒5)	4 (3‒4)	0.014^a^
Crew overall	5 (4‒5)	4.75 (4.75‒5)	4 (3‒4)	0.025^a^

Technical skills: adapted version of the survey developed and validated by Gaba et al.^16^

Non-technical skills: adapted version of the Line/LOS checklist developed by NASA (National Aeronautics and Space Administration) and the Aerospace Crew Performance Project. adapted by Gaba et al.^16^

a Kruskal-Wallis.

The mean score for non-technical skills was in all groups above 80% of the maximum of 60 (Table 3). A statistically significant lower score for non-technical skills was found for the control groups compared with the poster and application groups (Table 3). No significant difference was found between the groups that used cognitive aids (poster and application) (Table 3, Table 4). However, among the specific items in the non-technical skills survey, there was a significantly higher score of these groups compared to the control group in nine of the 12 items (Table 4). The effect size of poster and mobile application groups compared with the control group was considered large both for the technical (Cohen's *d* = 1.368, common language effect size 0.833) and for the non-technical skills (Cohen's *d* = 1.299 and 3.147, common language effect size 0.821 and 0.987, respectively).

The mean time to complete the scenario was 30 (7), 28 (11), and 25 (2) min for group control, poster, and application, respectively (p = 0.48).

## Discussion

This study assessed the performance of 15 pairs of residents in a realistic MH simulation. The perceived stress levels of participants were higher during the simulation, with no statistically significant difference detected between the groups at the three moments ‒ before, during, and after they participated in the simulation. Technical and non-technical skills were considered satisfactory. No pair neglected the essential items during the treatment of MH, which would have considerably affected their total score. When the three groups were compared regarding technical and non-technical skills, significantly higher scores were observed in the two groups that accessed the cognitive aids (either poster or application) compared to the control group.

The general technical skill score (fraction of the maximum possible score) was 87%, with the control group achieving a score of 81%. The study by Gaba et al. reported a technical skill score of 81%, meaning that, in this study, the appropriate technical actions were implemented. Gaba et al. also reported that none of the participants forgot essential items, a similar finding to that observed in this study.^16^

The non-technical skill scores observed in this study were higher than those reported by Gaba et al., where only 2 out of 14 (14%) participants had average scores equal to or higher than 4. In this study, the median non-technical skill score (average of the 12 survey items) was higher than 4 for all the pairs of residents.^16^ This could be explained by the fact that, currently, there is more available information about simulation-based training compared to when Gaba et al. performed their study. Also, our participants were anesthesiology residents. A study assessing the performance of board-certified anesthesiologists in simulated medical emergencies found an association between younger age and higher-rated performances.^18^

Previous studies using checklists to address MH cases have reported improved technical and behavioral performance.^19^ Despite the consensus regarding the usefulness of cognitive aids in managing crises, particularly during rare events, training and the continuous use of these tools are necessary.^20^ Our findings highlight the usefulness of both electronic and paper emergency manuals as cognitive aids, emphasizing the need for greater availability of these tools in the operating room.

Regarding technical and non-technical skills, no difference was observed between the poster and the application groups, suggesting that the content of both cognitive aids is similar. A hypothetical difference between the groups could be explained by the format of the information presented. Nevertheless, since the sample of this study was small, it was not possible to conclude whether one cognitive aid was better than the other. No previous studies testing an MH mobile application as a cognitive aid in an MH simulation were found. However, Sondheim et al. demonstrated the usefulness of a mobile application in the treatment of cardiac arrest in a prehospital care setting. The teams recorded more interventions, reported greater comfort, and reduced the number of errors as the number of team members increased to over three people.^24^

This study has some limitations. The residents were included sequentially, without a process of randomization. However, there were no differences among the three groups regarding pre-simulation stress or characteristics such as sex/age/previous experience with realistic simulation or MH that could systematically influence the results. We have not assessed the baseline skills among the participants, which may have affected the results. However, each pair of residents comprised one junior resident and one senior resident. To enhance the consistency of pre-intervention knowledge, all the participants attended a 2-hour class about MH.

This study is based on a realistic high-fidelity simulation scenario and some details regarding the quality of assistance could not be evaluated. For instance, Gardi et al. evaluated the quality of the hyperventilation performed by the participants through tidal volume, fresh gas flow, and respiratory rate. They observed that more than half of the participants carried it out improperly.^9^ In addition, the adopted scores have a well-known limitation: certain skills might exhibit variations throughout the simulation. To mitigate this issue, two independent observers were invited to score the pairs, which led to a consensual value. Notwithstanding, both technical and non-technical skill surveys were validated, reliable, and useful in a MH simulation.^16^ Finally, the small sample size and absence of longitudinal outcomes may affect the external validity of this study and the improved performance with cognitive aids should be interpreted while considering this limitation.

Simulation training, enhanced by cognitive aids, should be encouraged during resident training, continuous education programs in Anesthesiology, and MH crisis preparedness globally. Specifically in Brazil, many Medical schools have simulation centers that are open to many specialties and the Anesthesiology Society has an active simulation center. Considering the potential positive impact of incorporating cognitive aids into simulation-based curricula, the practical implications include the need to disseminate information about the checklists and mobile applications available in each situation, as well the initiative of teaching professionals how to use these tools to maximize their impact on clinical practice. Recommendations include explaining the format of the cognitive aid; familiarizing professionals with the layout, structure, and formatting; planning team use; reviewing items; using them in simulation; and conducting periodic reviews.^21^ Cognitive aids are particularly important in rare events such as MH, where their use is especially recommended by the European^22^ and North American^23^ MH associations.

Although simulation and cognitive aids have well-recognized positive impacts on education and training, longitudinal studies are needed to evaluate medium and long-term retention of skills. In addition, multi-institutional studies are required to validate these findings in diverse training environments.

## Conclusion

In a realistic high-fidelity MH simulation, the participants had satisfactory performance regarding technical and non-technical (behavioral) skills. The groups that had access to cognitive aids achieved better scores, but there was no difference between the group with access to the MH poster and the group with access to the MH mobile application. These findings underscore the potential benefits of simulation training, enhanced by cognitive aids, that can be encouraged both during resident training and continuous education programs in Anesthesiology*.*

## Declaration of competing interest

The authors declare no conflicts of interest.

## Appendix Supplementary materials

Image, application 1

## Footnotes

Supplementary material associated with this article can be found in the online version at doi:10.1016/j.bjane.2025.844615.
