# Supplementary material for: Evaluation of anesthesiology residents in the diagnosis and control of malignant hyperthermia: comparison of three scenarios of realistic simulation ‒ a cross-sectional controlled study
Source: Braz J Anesthesiol. 2025 Mar 28;75(4):844615. doi: 10.1016/j.bjane.2025.844615 (PMC12008681; doi:10.1016/j.bjane.2025.844615)
Supplement: Supplementary file 1 [file mmc1.docx]

**BJAN-D-24-00496_ Supplementary Material**

**Supplementary** **Material 1 Table 1**


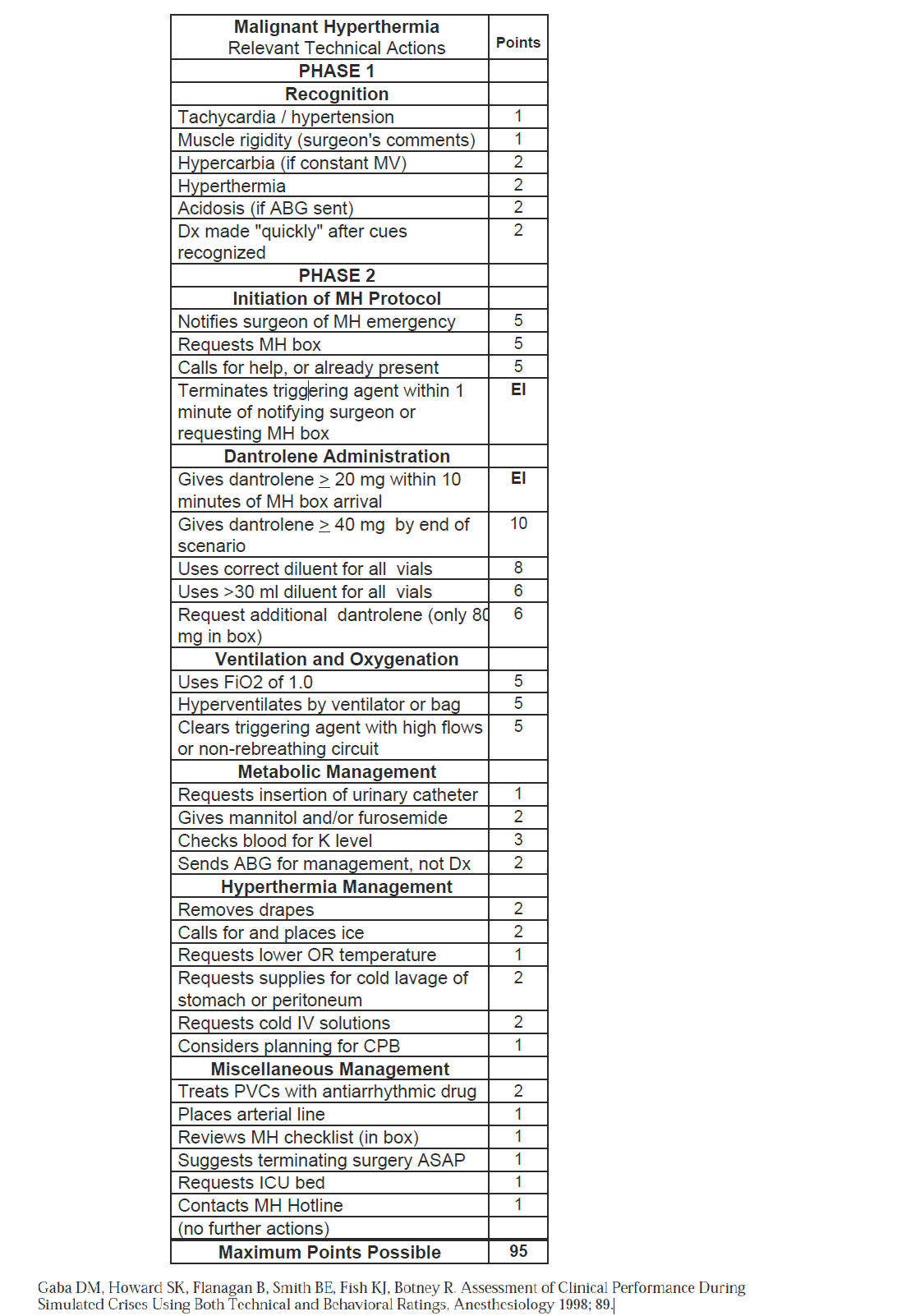


**Supplemental Material Table 2**

Scenario name_____________ Tape ID or date_____________ Rater & date_____________

Crisis management behavioral performance markers.

| 0 | 1 | 2 | 3 | | 4 | | | | 5 | | | |
| --- | --- | --- | --- | --- | --- | --- | --- | --- | --- | --- | --- | --- |
| Not Observed (ALSO select a rating from 1‒5) | Poor Performance | Minimally Acceptable Performance | Standard Performance | | Good Performance | | | | Excellent Performance | | | |
| **1b. Orientation** | | | | **Phase 1:** | | **0** | **1** | **2** | | **3** | **4** | **5** |
| Introduces oneself or enters flow of case | | | |  |  |  |  |  |  |  |  |  |
| Makes specific observations of patient status | | | |  |  |  |  |  |  |  |  |  |
| Seeks information from personnel and/or records | | | |  |  |  |  |  |  |  |  |  |
| Checks the operation of anesthesia equipment | | | |  |  |  |  |  |  |  |  |  |
| Comments: | | | |  |  |  |  |  |  |  |  |  |
| **2. Inquiry/Advocacy/Assertion** | | | | **Phase 1:** | | **0** | **1** | **2** | | **3** | **4** | **5** |
| Inquiry is encouraged, & questions answered openly | | | |  |  |  |  |  |  |  |  |  |
| Crew members seek info from others & speak up with appropriate persistence | | | |  |  |  |  |  |  |  |  |  |
| Someone other than the main anesthetist assumes command when necessary | | | |  |  |  |  |  |  |  |  |  |
| Anesthetist(s) insists on suspension of surgery with appropriate assertiveness | | | |  |  |  |  |  |  |  |  |  |
| Comments: | | | |  |  |  |  |  |  |  |  |  |
| **3. Communications** | | | | **Phase 1:** | | **0** | **1** | **2** | | **3** | **4** | **5** |
| Crew members notify each other of necessary info | | | |  |  |  |  |  |  |  |  |  |
| Messages stated precisely and to specific individuals | | | |  |  |  |  |  |  |  |  |  |
| Crew members acknowledge communications and verify ambiguous communications | | | |  |  |  |  |  |  |  |  |  |
| Efforts are made to establish and maintain an open atmosphere | | | |  |  |  |  |  |  |  |  |  |
| Tone of voice is appropriate to the situation | | | |  |  |  |  |  |  |  |  |  |
| Comments: | | | |  |  |  |  |  |  |  |  |  |
| **4. Feedback** | | | | **Phase 1:** | | **0** | **1** | **2** | | **3** | **4** | **5** |
|  |  |  |  | **Phase 2:** | | **0** | **1** | **2** | | **3** | **4** | **5** |
| Errors in mgmt are identified & corrected | | | |  |  |  |  |  |  |  |  |  |
| Feedback addresses + as well as - performance. | | | |  |  |  |  |  |  |  |  |  |
| Is given & accepted objectively and nondefensively. | | | |  |  |  |  |  |  |  |  |  |
| Is given in appropriate manner & times | | | |  |  |  |  |  |  |  |  |  |
| Comments: | | | |  |  |  |  |  |  |  |  |  |
| **5. Leadership/Followership** | | | | **Phase 1:** | | **0** | **1** | **2** | | **3** | **4** | **5** |
| **Note: This marker addresses the overall performance of the crew as a whole** | | | | **Phase 2:** | | **0** | **1** | **2** | | **3** | **4** | **5** |
| Leader: | | | |  |  |  |  |  |  |  |  |  |
| The “hot seat” anesthetist takes command or delegates command to more qualified associate | | | |  |  |  |  |  |  |  |  |  |
| Help is called for as necessary; errs on side of calling for help | | | |  |  |  |  |  |  |  |  |  |
| The leader acts decisively (e.g., commits to declare emergency early vs. late) | | | |  |  |  |  |  |  |  |  |  |
| Coordinates activities of all crew; checks with crew about task status | | | |  |  |  |  |  |  |  |  |  |
| Stays free to direct except when necessary. | | | |  |  |  |  |  |  |  |  |  |
| Followers: | | | |  |  |  |  |  |  |  |  |  |
| Identify the leader clearly | | | |  |  |  |  |  |  |  |  |  |
| Respond promptly; report task status periodically | | | |  |  |  |  |  |  |  |  |  |
| Work through leader most of time; exert leadership as necessary to backup “hot seat” anesthetist | | | |  |  |  |  |  |  |  |  |  |
| Comments: | | | |  |  |  |  |  |  |  |  |  |
| **6. Group climate** | | | | **Phase 1:** | | **0** | **1** | **2** | | **3** | **4** | **5** |
|  |  |  |  | **Phase 2:** | | **0** | **1** | **2** | | **3** | **4** | **5** |
| Crew and team members remain calm. Innapropriate behaviors are ignored or countered properly | | | |  |  |  |  |  |  |  |  |  |
| Atmosphere in the OR is relaxed but escalates as appropriate; group attention is focused on patient care | | | |  |  |  |  |  |  |  |  |  |
| Distractions are actively modulated (e.g. music is turned down or off when workload increases). | | | |  |  |  |  |  |  |  |  |  |
| Comments: | | | |  |  |  |  |  |  |  |  |  |
| **7. Preparation/Planning/Anticipation** | | | | **Phase 1:** | | **0** | **1** | **2** | | **3** | **4** | **5** |
|  |  |  |  | **Phase 2:** | | **0** | **1** | **2** | | **3** | **4** | **5** |
| Crew and team members are made aware of important plans | | | |  |  |  |  |  |  |  |  |  |
| Crew members identify milestones, abort points, and contingency plans & are ready for them | | | |  |  |  |  |  |  |  |  |  |
| Crew members prepare to escalate to more aggressive or complex therapies | | | |  |  |  |  |  |  |  |  |  |
| Event follow up takes place | | | |  |  |  |  |  |  |  |  |  |
| Comments: | | | |  |  |  |  |  |  |  |  |  |
| **8. Workload distribution** | | | | **Phase 1:** | | **0** | **1** | **2** | | **3** | **4** | **5** |
|  |  |  |  | **Phase 2:** | | **0** | **1** | **2** | | **3** | **4** | **5** |
| Crew or team members manage problems and execute tasks with appropriate priority. | | | |  |  |  |  |  |  |  |  |  |
| Tasks are assigned to specific, appropriately trained individuals | | | |  |  |  |  |  |  |  |  |  |
| Help is called for, when appropriate (errs on side of calling for help). | | | |  |  |  |  |  |  |  |  |  |
| Crew or team members report work overloads and recognize and report work overloads in others. | | | |  |  |  |  |  |  |  |  |  |
| Comments: | | | |  |  |  |  |  |  |  |  |  |
| **9. Vigilance** | | | | **Phase 1:** | | **0** | **1** | **2** | | **3** | **4** | **5** |
|  |  |  |  | **Phase 2:** | | **0** | **1** | **2** | | **3** | **4** | **5** |
| Demonstrates awareness of special characteristics of patient or situation. | | | |  |  |  |  |  |  |  |  |  |
| Monitors & cross-checks all sources of information | | | |  |  |  |  |  |  |  |  |  |
| Considers abnormalities to be real until proven to be false | | | |  |  |  |  |  |  |  |  |  |
| Comments: | | | |  |  |  |  |  |  |  |  |  |
| **10. Reevaluation** | | | | **Phase 1:** | | **0** | **1** | **2** | | **3** | **4** | **5** |
|  |  |  |  | **Phase 2:** | | **0** | **1** | **2** | | **3** | **4** | **5** |
| Reevaluates the results and side effects of interventions or actions. | | | |  |  |  |  |  |  |  |  |  |
| Advances to more aggressive interventions, when initial therapy is unsuccessful. | | | |  |  |  |  |  |  |  |  |  |
| Information & mental models are shared with crew | | | |  |  |  |  |  |  |  |  |  |
| Avoids fixation errors | | | |  |  |  |  |  |  |  |  |  |
| Comments: | | | |  |  |  |  |  |  |  |  |  |
| **11. Overall HOT-SEAT PERSON effectiveness** | | | | **Phase 1:** | | **0** | **1** | **2** | | **3** | **4** | **5** |
|  |  |  |  | **Phase 2:** | | **0** | **1** | **2** | | **3** | **4** | **5** |
| **12 Overall ANESTHESIA CREW effectiveness** | | | | **Phase 1:** | | **0** | **1** | **2** | | **3** | **4** | **5** |
|  |  |  |  | **Phase 2:** | | **0** | **1** | **2** | | **3** | **4** | **5** |
| Comments: | | | |  | |  |  |  | |  |  |  |
| Comments about the conduct of the simulation scenario or additional comments or information: | | | | | | | | | | | | |

Gaba DM, Howard SK, Flanagan B, Smith BE, Fish KJ, Botney R. Assessment of Clinical Performance. During Simulated Crises Using Both Technical and Behavioral Ratings, Anesthesiology 1998;89.

**Supplemental Material Table 3** Spearman correlation analysis between stress levels and performance (technical and non-technical) at each time point (before/during/after simulation).

|  | **Correlation coefficient (Spearman)** | **p** |
| --- | --- | --- |
| **Stress before worst score of the pair X TS** | -0.3 | 0.278 |
| **Stress before average score of the pair X TS** | -0.39 | 0.150 |
| **Stress during worst score of the pair X TS** | -0.128 | 0.650 |
| **Stress during average score of the pair X TS** | 0.029 | 0.918 |
| **Stress after worst score of the pair X TS** | -0.216 | 0.438 |
| **Stress after average score of the pair X TS** | -0.110 | 0.697 |
| **Stress before worst score of the pair X NTS** | 0.041 | 0.886 |
| **Stress before average score of the pair X NTS** | -0.055 | 0.845 |
| **Stress during worst score of the pair X NTS** | 0.133 | 0.635 |
| **Stress during average score of the pair X NTS** | 0.246 | 0.376 |
| **Stress after worst score of the pair X NTS** | 0.160 | 0.569 |
| **Stress after average score of the pair X NTS** | 0.183 | 0.513 |

TS, Technical Skills; NTS, Non-Technical Skills.
